# Prognostic and Clinicopathological Value of ZWINT Expression Levels in Patients with Lung Adenocarcinoma: A Systematic Review and Meta-analysis

**DOI:** 10.6061/clinics/2021/e3222

**Published:** 2021-11-17

**Authors:** Ran Zhu, Huaguo Wang, Ling Lin

**Affiliations:** I Department of Clinical Laboratory, The First People’s Hospital of Ziyang, Sichuan, China.; II Department of Respiratory and Critical Care Medicine, The First People’s Hospital of Ziyang, Sichuan, China.

**Keywords:** Prognosis, ZWINT, Lung Adenocarcinoma, Meta-Analysis, TCGA

## Abstract

The current study found that high Zeste White 10 interactor (ZWINT) expression is related to the poor prognosis of patients with a variety of cancers. This study mainly explored the relationship between the expression level of ZWINT and the prognosis of patients with lung adenocarcinoma (LUAD). Briefly, four English databases and two high-throughput sequencing databases were searched and relevant data for meta-analysis were extracted. Pooled mean difference and 95% confidence interval (CI) were used to assess the relationships between clinical features and the expression of ZWINT. Pooled hazard ratio and 95% CI were also used to assess the relationships between clinical features and the expression level of ZWINT. This meta-analysis was registered in PROSPERO (CRD42021249475). A total of 16 high-quality datasets comprising 2,847 LUAD patients were included in this study. Higher ZWINT expression levels were found in patients younger than 65 years, males, and smokers, and were correlated with advanced TNM stages and poor prognosis. Notably, there was no publication bias in this meta-analysis. Overall, our findings indicate that ZWINT is a potential biomarker for poor prognosis and clinicopathological outcomes of patients with LUAD.

## INTRODUCTION

### Background

Cancer is the leading cause of death and the reduction in life expectancy on a global scale. The burden of cancer as well as cancer incidence and mortality rates are increasing rapidly worldwide (1). In 2020, an estimated 19.3 million new cancer cases were diagnosed globally (18.1 million excluding nonmelanoma skin cancers), with nearly 10 million deaths (9.9 million excluding nonmelanoma skin cancers). Breast cancer has surpassed lung cancer as the most common cancer in women; however, lung cancer remains the leading cause of cancer-related death (1). In China, lung cancer is also the leading cause of cancer death and morbidity for both men and women (2). Of the pathological types of lung cancer, most are lung adenocarcinoma (LUAD) (3).

Zeste White 10 interactor (ZWINT) is an important protein that regulates centromeric division, playing a key role in chromosomal motion and mitosis (4). ZWINT is significantly overexpressed in a variety of cancers and is closely associated with the prognosis of patients with these cancers. Previously, ZWINT knockout was found to inhibit the migration, apoptosis, and colony formation of cancer cells while its downregulation reduced tumor volume. High ZWINT expression was also demonstrated to be closely related to the poor prognosis of patients with LUAD (5). Shorter relapse-free survival, overall survival (OS), and metastatic relapse-free survival may also be associated with higher ZWINT expression in patients with breast cancer (6).

With the advent of precision medicine and the development of sequencing technology, advancements in personalized genomics research have occurred because of the development of individual protocols for cancer and other diseases based on a person's genetic information (7,8). Moreover, the medical model is gradually changing from empirical-based to an evidence-based model. At present, evidence-based medicine is mainly assessed via systematic evaluation and meta-analysis (9). Meta-analysis is a type of systematic evaluation in which data are statistically processed by quantitative synthesis, termed quantitative systematic evaluation. The greatest advantage of meta-analysis is that it avoids the limitation of a single small-sample clinical trial and can evaluate controversial results and resolve contradictions between studies, thereby providing good evidence for clinical decision making (10). In this study, we conducted a meta-analysis to clarify the prognostic and clinicopathological value of ZWINT in LUAD.

## MATERIALS AND METHODS

### Study Search

PubMed, EMBASE, Web of Science, and Cochrane Library were searched and the medical subject headings of LUAD and ZWINT were defined according to https://www.ncbi.nlm.nih.gov/mesh/. Data from two high-throughput sequencing databases, namely The Cancer Genome Atlas (TCGA) and Gene Expression Omnibus (GEO), were also retrieved for subsequent analysis. The search terms for PubMed were ((((Lung Adenocarcinomas) OR (Lung Adenocarcinoma)) OR (Adenocarcinoma, Lung)) OR (Adenocarcinomas, Lung)) AND ((((((ZW10 interacting protein-1) OR (Zwint1 protein)) OR (ZW10 interactor)) OR (Zwint-1 protein)) OR (ZW10 interacting protein 1)) OR (ZW10 interactor protein)). The retrieval method was adjusted according to the characteristics of the database and each database was searched from its inception to May 1, 2021. The languages of publications were limited to English and Chinese. As computer retrieval was limited by the literature and the indexing and retrieval strategy of the database itself, the recall and precision of the results may be affected. Therefore, in addition to computer retrieval, we manually searched all references in the original studies to ensure that all eligible studies were included.

### Inclusion Criteria

The criteria for study inclusion were as follows: 1. included the relationship between ZWINT expression and LUAD prognostic indicators, such as OS and progression-free survival (PFS); 2. included corresponding statistical indicators, such as the hazard ratio (HR) and 95% confidence interval (CI), and if the HR and 95% CI were not clearly reported, the corresponding values could be calculated according to the information provided in the study; 3. included the most complete or latest study with the same research results; and 4. included human subjects. Nonoriginal studies, such as reviews, meta-analyses, case reports, and comments, were excluded from the analysis.

### Selection and Inclusion Processes

Evidently repeated publications were first removed through a literature search, and publications that were obviously unrelated to this study were excluded via careful reading of the titles and abstracts. The full texts of studies that potentially met the inclusion criteria were examined. Different results for the same study were integrated, and the full text of the selected literature was carefully read to determine whether it met the inclusion criterion of an original article for the systematic review. When the relevant information needed in the literature research was incomplete or unclear, this was either obtained by reasonable deduction from the literature or clarified by the corresponding author. Finally, we decided whether the study should be included. For TCGA and GEO data, we also obtained relevant literature and screened them using a similar approach.

### Quality Evaluation

To avoid bias in the quality evaluation, two reviewers assessed the included studies. The two reviewers independently used the Newcastle-Ottawa Scale (NOS) to evaluate quality (11), and communication and negotiation were carried out accordingly to ensure consistent application of the evaluation standards. The quality of the literature was evaluated formally. If the selection and evaluation of the literature differed between reviewers, a discussion was held or relevant professional researchers were asked to evaluate the studies.

### Data Extraction

Two authors independently conducted literature quality assessment and data extraction, and then performed an in-depth reading of the text. Studies were then selected according to the above inclusion criteria. During the data extraction process, the data extracted by one reviewer were regularly checked by the other to identify differences in a timely manner. In the event of different decisions, a judgment was made by joint discussion or with the help of other professional researchers.

We downloaded RNA-seq data from TCGA in the fragments per kilobase of exon model per million mapped fragments (FPKM) format from the official website (https://portal.gdc.cancer.gov/) and converted the data to the ENSG ID gene symbol. We also downloaded clinical data for patients with LUAD. For the GEO database (https://www.ncbi.nlm.nih.gov/geo/), we downloaded raw microarray expression matrix data and converted the probe name to the gene symbol according to different sequencing platforms using the limma package and log2 translation (12). If more than one probe was used, the average value was employed. The corresponding clinical information was also downloaded.

### Meta-analysis

Upon comparing the expression level of ZWINT with different clinical features, the standard mean difference (SMD) and 95% CI were used as statistics for the combined analysis. When the correlation between expression level of ZWINT and prognosis was investigated, the HR and 95% CI were used as statistics for the combined analysis. The Q and I^2^ tests were used to assess heterogeneity. Values of *p*<0.05 and I^2^>50% indicated high heterogeneity among the studies; the random effect model was used for meta-analysis. Values of *p*>0.05 and I^2^<50% indicated no or low heterogeneity among the studies; a fixed effect model was employed for this analysis. The results are presented as a forest map. By eliminating one study at a time, the remaining studies were combined to assess the degree of change in the results of sensitivity analysis. Begg’s test was used to evaluate publication bias.

### Construction and validation of the nomogram of ZWINT expression

TCGA database has the most detailed information about the clinical characteristics and follow-up of LUAD patients. Therefore, we used TCGA data to construct a nomogram containing ZWINT expression levels to prove the clinical application value of ZWINT. Xtile software (version 3.6.1) was used to derive the best cutoff value of ZWINT and divide patients into high expression and low expression groups. The principle is to group different values as cutoff values for statistical tests. The result with the smallest *p* value can be considered the best cutoff value. Kaplan-Meier curves and log rank tests were used to detect the difference in prognosis between the high and low ZWINT expression level groups.

We combined the clinical information of patients and the expression level of ZWINT for multivariate Cox regression analysis. For statistically significant factors (*p*<0.05), a nomogram was generated. In the survival analysis, the disease status and factor values will change with time. Accordingly, the use of a time-dependent ROC curve is undoubtedly a better choice. Therefore, a time-dependent ROC curve was used to determine the predictive ability of the nomogram. In addition, a calibration curve was used to verify the predictive ability of the nomogram.

## RESULTS

### Literature and Dataset Search

**Figure 1:**
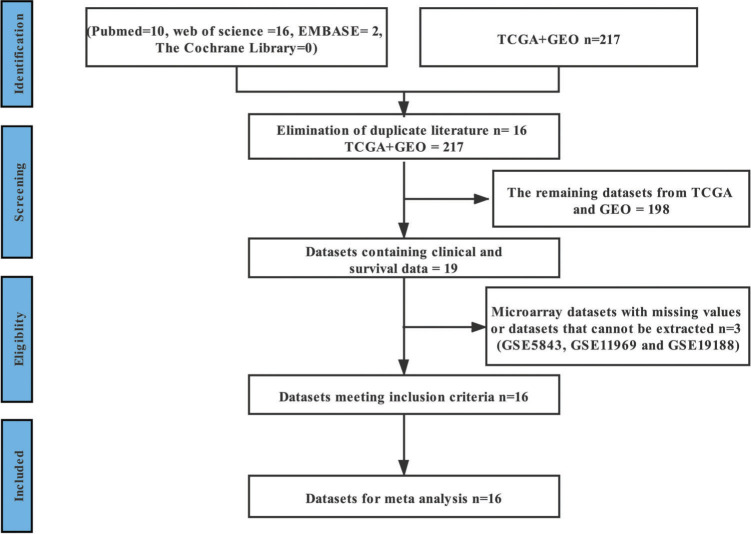
The general screening process for inclusion in the study.

After excluding repetitive studies, 65 relevant studies from four English databases were retained according to the inclusion and exclusion criteria. After careful reading of the 65 manuscripts, we found that no data could be extracted. However, in the two high-throughput sequencing databases, the following datasets were found to contain enough data for subsequent analysis: GSE3141 (13), GSE8894 (14), GSE13213 (15), GSE14814 (16), GSE26939 (17), GSE29013 (18), GSE30219 (19), GSE31210 (20), GSE37745 (21), GSE41271 (22), GSE42127 (23), GSE50081 (24), GSE68465 (25), GSE72094 (26), GSE83227 (27), and TCGA (28). The process of document retrieval and inclusion is shown in Figure 1. The basic information of the 16 datasets is presented in Table 1. The data of 2,847 patients with LUAD were included in this study and the clinical characteristics of these patients are shown in Table S1. According to the literature corresponding to these databases, these studies are of high quality and have high NOS scores (Table 2). This meta-analysis was registered in PROSPERO (CRD42021249475).

### Associations Between ZWINT Expression and Clinical Characteristics

**Figure 2:**
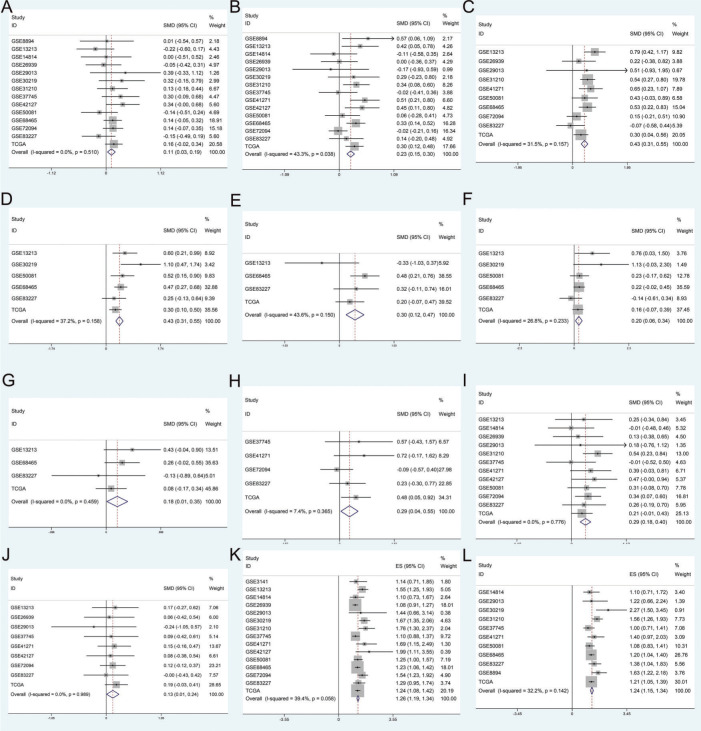
The results of the meta-analysis for the association of ZWINT expression with patient (**A**) age (<=65/>65), (**B**) sex (male/female), (**C**) smoking status (yes/no), (**D**) T stage (T2/T1), (**E**) T stage (T3-T4/T1-T2), (**F**) N stage (N1/N0), (**G**) N stage (N2-N3/N0-N1), (**H**) M stage (M1/M0), (**I**) AJCC stage (stage II/stage I), (**J**) AJCC stage (stage III-IV/stage I-II), (**K**) OS, and (**L**) PFS.

The results of the association analyses of ZWINT expression and clinical characteristics are shown in Table 3 and Figure 2A-J. The expression level of ZWINT was higher in patients younger than 65 years than in patients older than 65 years (SMD=0.109, 95% CI=0.028 and 0.190, *p*=0.009). Male patients had higher ZWINT expression levels than female patients (SMD=0.198, 95% CI=0.133 and 0.262, *p*<0.001). The expression levels of ZWINT were higher in patients with a history of smoking than in nonsmoking patients (SMD=0.428, 95% CI=0.310 and 0.545, *p*<0.001). The expression of ZWINT was also higher in patients with higher TNM stages as depicted by the following statistics: T stage (T2/T1: SMD=0.428, 95% CI=0.310 and 0.545, *p*<0.001; T3-T4/T1-T2: SMD=0.295, 95% CI=0.124 and 0.466, *p*=0.001), N stage (N1/N0: SMD=0.199, 95% CI=0.057 and 0.341, *p*=0.006; T3-T4/T1-T2: SMD=0.183, 95% CI=0.011 and 0.355, *p*=0.037), M stage (M1/M0: SMD=0.293, 95% CI=0.036 and 0.550, *p*=0.025), and AJCC stage (II/I: SMD=0.287, 95% CI=0.178 and 0.396, *p*<0.001; III-IV/I-II: SMD=0.126, 95% CI=0.008 and 0.243, *p*=0.036). The above meta-analysis was based on a fixed effect model because of zero or low heterogeneity.

### Association of ZWINT Expression with Prognosis

High ZWINT expression levels indicated worse OS and PFS for LUAD patients. Fifteen datasets contained survival data, which could be used to calculate the OS; the pooled results were HR=1.263, 95% CI=1.187-1.340, and *p*<0.001 with low heterogeneity using a fixed effect model. Eleven datasets contained survival data, which could be used to calculate the PFS; the pooled results were HR=1.243, 95% CI=1.150-1.336, and *p*<0.001 with low heterogeneity using a fixed effect model. The results are shown in Figure 2K and L, and Table 1.

### Sensitivity Analyses and Publication Bias

**Figure 3:**
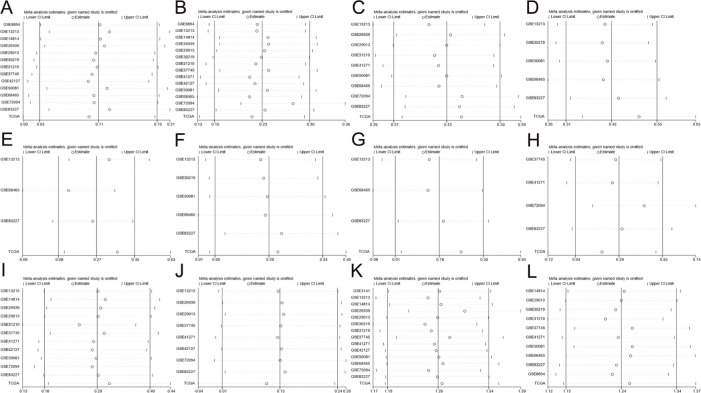
Sensitivity analyses of patient (**A**) age (<=65/>65), (**B**) sex (male/female), (**C**) smoking status (yes/no), (**D**) T stage (T2/T1), (**E**) T stage (T3-T4/T1-T2), (**F**) N stage (N1/N0), (**G**) N stage (N2-N3/N0-N1), (**H**) M stage (M1/M0), (**I**) AJCC stage (stage II/stage I), (**J**) AJCC stage (stage III-IV/stage I-II), (**K**) OS, and (**L**) PFS.

**Figure 4:**
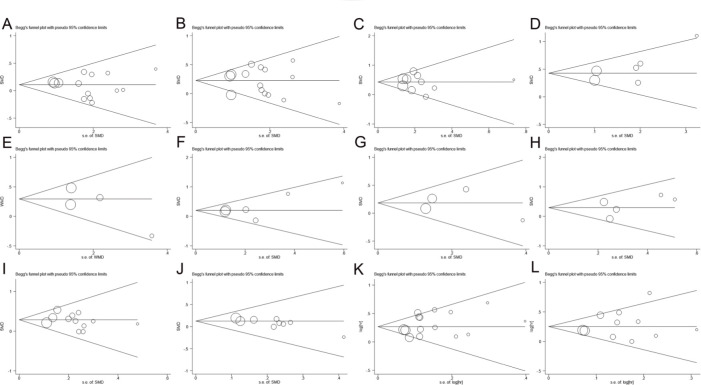
The results of publication bias for patient (**A**) age (<=65/>65), (**B**) sex (male/female), (**C**) smoking status (yes/no), (**D**) T stage (T2/T1), (**E**) T stage (T3-T4/T1-T2), (**F**) N stage (N1/N0), (**G**) N stage (N2-N3/N0-N1), (**H**) M stage (M1/M0), (**I**) AJCC stage (stage II/stage I), (**J**) AJCC stage (stage III-IV/stage I-II), (**K**) OS, and (**L**) PFS.

As shown in Figure 3, no study was found to significantly affect the total pooled results alone, suggesting that this meta-analysis provided reliable results. As shown in Figure 4 and Table 1, no significant publication bias was found among all studies.

### Construction and validation of the nomogram of ZWINT expression

**Figure 5:**
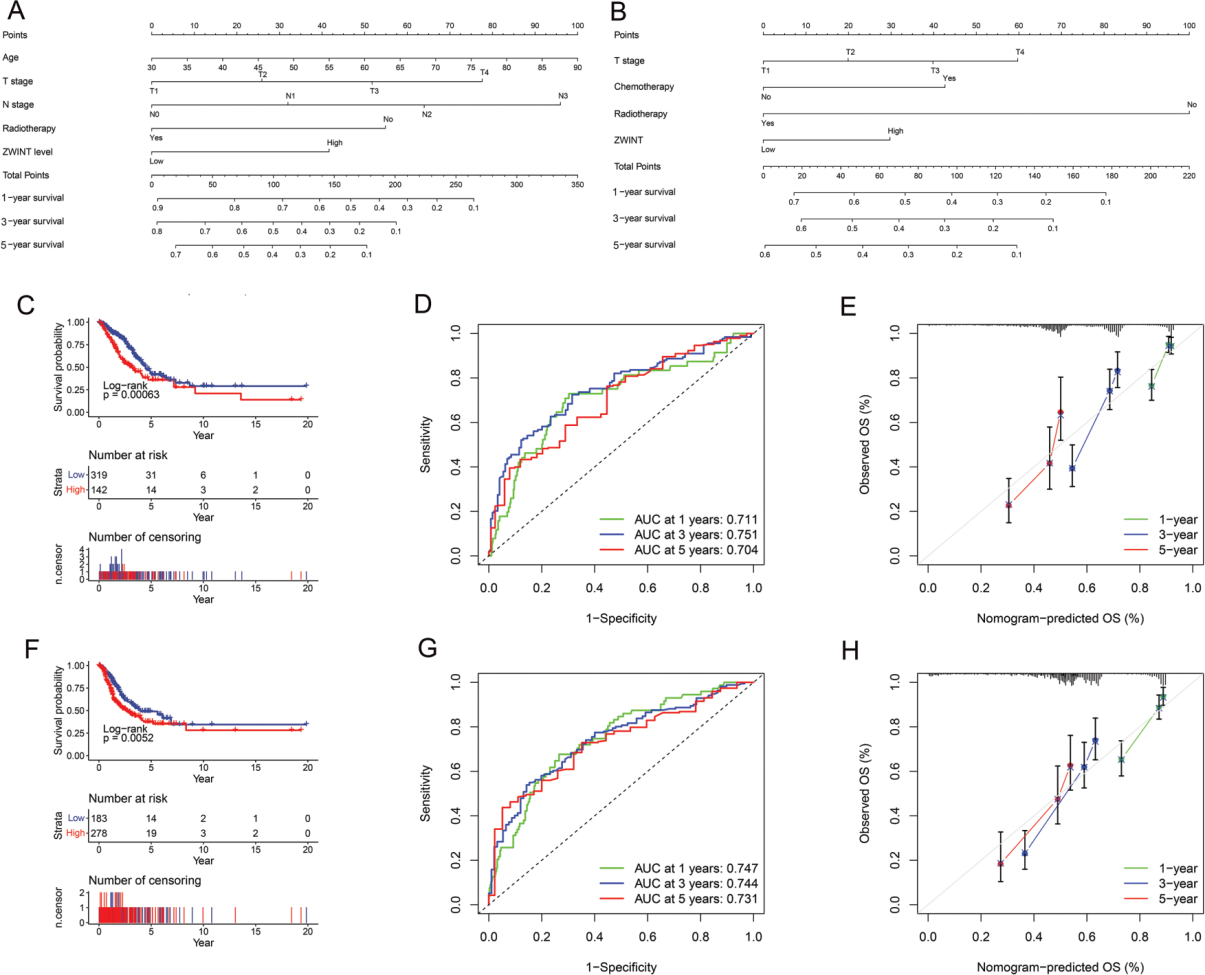
Nomogram of the association between ZWINT expression level and OS (A) and PFS (B). (C). Kaplan-Meier survival curve depicting the OS of patients with different ZWINT expression levels. (D). Time-dependent ROC curve of OS. (E). Calibration curve of OS. (F). Kaplan-Meier survival curve depicting the PFS of patients with different ZWINT expression levels. (G). Time-dependent ROC curve of PFS. (H). Calibration curve of PFS.

Significant differences in OS (Figure 5C) and PFS (Figure 5F) were found between the high and low ZWINT expression groups. Multivariate Cox regression analysis also revealed that age, T stage, N stage, receiving radiotherapy, and ZWINT expression were independent prognostic factors for OS, while T stage, receiving radiotherapy, receiving chemotherapy, and ZWINT expression were independent prognostic factors for PFS (Table S2). The nomograms for predicting OS and PFS are shown in Figure 5A and 5B. The area under the curve (AUC) of the nomogram for OS at 1 year, 3 years, and 5 years was 0.711, 0.751, and 0.704 (Figure 5D), respectively, while that for PFS at 1 year, 3 years, and 5 years was 0.747, 0.744, and 0.731 (Figure 5G), respectively. The calibration curve (Figure 5E and 5H) also suggested that our nomogram has great prediction ability.

## DISCUSSION

In this study, we extensively searched four major English databases and two high-throughput sequencing databases; however, no literature reports on ZWINT and the prognosis of patients with LUAD were available. Nonetheless, in two high-throughput sequencing databases, we found datasets related to ZWINT expression and LUAD. Using the data in these datasets, we conducted a meta-analysis of individual patient data (IPD). The results of the IPD meta-analysis indicated significantly high ZWINT expression in patients younger than 65 years old, men, smoking patients, and patients with higher TNM stages. This study confirmed that ZWINT is related to the prognosis of LUAD patients. Further analysis revealed that the positive expression of ZWINT in LUAD was closely related to TNM stage. The higher the TNM stage, the higher the positive expression rate of ZWINT, which suggested that ZWINT might be involved in the occurrence, development, invasion, and metastasis of LUAD. However, the regulatory mechanism and function of ZWINT expression in esophageal cancer are not completely clear. Therefore, ZWINT may be used as a biomarker for predicting poor clinicopathology and the prognosis of patients with LUAD.

The protein encoded by *ZWINT* is composed of 278 amino acids, and plays a regulatory role in the cell cycle (29). Previously, ZWINT was found to be related to chromosome instability, which promotes the occurrence and development of a variety of malignant tumors (30). ZWINT has also been found to be related to the occurrence and development of a variety of tumors. For example, Wang et al. (31) used bioinformatics to investigate and analyze the differences in gene expression between normal and nasopharyngeal carcinoma tissues and found that significantly high ZWINT expression was related to nasopharyngeal carcinoma tissues. Akabane et al. (32) found that KIFC1 was positive in 67 (52%) of 129 patients with colorectal cancer based on immunohistochemistry; this positivity was also found to be related to the low OS rate. Moreover, the expression of ZWINT was found to be significantly correlated with KIFC1 expression, and *KIFC1* and *ZWINT* knockout cells were observed to reduce the tumor formation ability (32). Kim et al. (33) found that the invasion and migration abilities of ZWINT-deficient pancreatic cancer cells were decreased; the expression levels of MMP2 and MMP9 were decreased; and the cell cycle arrested in the G2/M phase. The apoptosis rate was also gradually increased, and was accompanied by caspase-3 activation and anti-poly (ADP ribose) polymerase cleavage (33). The relative level of ZWINT expression decreased gradually with the progression of the cell cycle and decreased sharply during mitotic withdrawal. Treatment with cycloheximide reduced the level of ZWINT, while treatment with MG132 to inhibit the endogenous ubiquitin proteasome increased the level of ZWIN-1 in HEK293T cells and HeLa cells. These data suggest that ZWINT may be degraded by the endogenous ubiquitin proteasome (34).

ZWINT is also related to the pathological mechanism of lung cancer and may serve as a new biomarker. Using qRT-PCR, Peng et al. found that ZWINT was markedly overexpressed in lung cancer tissue and that knocking out ZWINT could reduce the proliferation of ncih226 and A549 cells; inhibit the migration, invasion, apoptosis, and colony formation of cancer cells; and reduce the tumor volume (5). Further, these researchers combined the clinical and survival follow-up data from TCGA to confirm that high ZWINT expression is associated with poor prognosis in patients with LUAD but not in patients with lung squamous cell carcinoma (LUSC). Some studies have confirmed that ZWINT is not only related to the prognosis of LUAD, but can also be used as a biomarker for the diagnosis of early lung cancer with high sensitivity (35,36).

With the continuous development of high-throughput sequencing technology and precision medicine, an increasing number of studies are focusing on the relationships between genes and diseases, especially cancer (37) and other nontumor chronic diseases (38). The studies included in this meta-analysis were high-throughput sequencing analyses. Although this technology has many advantages, it is associated with a high cost, complex operation, and difficult clinical application. Notably, immunohistochemistry has the advantages of simple operation, low economic cost, localization, and characterization. Further, compared with other protein detection methods, immunohistochemistry provides more direct and accurate localization and has high qualitative sensitivity. Accordingly, it is the preferred method for localization detection and analyses, and is especially useful for the transposition of some factors. The current research supports the proposal that tumors are essentially genetic diseases (39,40). In fact, *ZWINT* could serve as a new biomarker for LUAD.

To the best of our knowledge, this is the first IPD meta-analysis of the relationship between ZWINT expression and LUAD prognosis. This IPD meta-analysis overcame the shortcomings of limited survival data, insufficient amounts of long-term follow-up data, and insufficient utilization of outcome indicators for each research object, while obtaining more accurate conclusions (41). However, our research has limitations. First, although we included a sufficient number of studies in this analysis, our overall sample size was still slightly small. Second, because of the large time span of the included studies, the TNM stages of some patients may have been determined based on different criteria. Finally, most of the research data were derived from Europe and the United States. Accordingly, data from countries with high cancer incidence rates, especially China and Asia, as well as a global representation, are insufficient and lacking.

## CONCLUSIONS

This meta-analysis revealed high ZWINT expression in young, male LUAD patients who smoke and have high TNM stages. Further, high ZWINT expression was found to be significantly associated with poor prognosis. However, such findings need to be further confirmed with a larger sample size and well-designed clinical trials.

## AUTHOR CONTRIBUTIONS

Zhu R and Wang H conceived and designed the study, acquired and analyzed the data, and wrote the manuscript. Lin L contributed to data analysis and manuscript preparation. All authors read and approved the final version of the manuscript and agree to be accountable for all aspects of the research with regard to accuracy or integrity.

## Figures and Tables

**Table 1:** Basic characteristics of the included datasets.

Datasets	First author	Year	Platform	No. of included LUAD patients	Country	PMID
GSE3141	Andrea H Bild	2005	GPL570 [HG-U133_Plus_2] Affymetrix Human Genome U133 Plus 2.0 Array	58	USA	16273092
GSE8894	Eung-Sirk Lee	2007	GPL570 [HG-U133_Plus_2] Affymetrix Human Genome U133 Plus 2.0 Array	63	South Korea	19010856
GSE13213	Shuta Tomida	2008	GPL6480 Agilent-014850 Whole Human Genome Microarray 4x44K G4112F (Probe Name version)	117	Japan	19414676
GSE14814	Chang-Qi Zhu	2009	GPL96 [HG-U133A] Affymetrix Human Genome U133A Array	71	Canada	20823422
GSE26939	Wilkerson MD	2011	GPL9053 Agilent-UNC-custom-4X44K	116	USA	22590557
GSE29013	Yang Xie	2011	GPL570 [HG-U133_Plus_2] Affymetrix Human Genome U133 Plus 2.0 Array	30	USA	21742808
GSE30219	Sophie Rousseaux	2011	GPL570 [HG-U133_Plus_2] Affymetrix Human Genome U133 Plus 2.0 Array	106	France	23698379
GSE31210	Hirokazu Okayama	2011	GPL570 [HG-U133_Plus_2] Affymetrix Human Genome U133 Plus 2.0 Array	226	Japan	23028479
GSE37745	Miriam Lohr	2012	GPL570 [HG-U133_Plus_2] Affymetrix Human Genome U133 Plus 2.0 Array	106	Sweden	26608184
GSE41271	Luc Girard	2012	GPL6884 Illumina HumanWG-6 v3.0 expression beadchip	181	USA	27354471
GSE42127	Hao Tang	2012	GPL6884 Illumina HumanWG-6 v3.0 expression beadchip	133	USA	23357979
GSE50081	Sandy D Der	2013	GPL570 [HG-U133_Plus_2] Affymetrix Human Genome U133 Plus 2.0 Array	181	Canada	24305008
GSE68465	Kerby Shedden	2015	GPL96 [HG-U133A] Affymetrix Human Genome U133A Array	442	USA	18641660
GSE72094	M B Schabath	2015	GPL15048 Rosetta/Merck Human RSTA Custom Affymetrix 2.0 microarray [HuRSTA_2a520709.CDF]	398	USA	26477306
GSE83227	A Bhattacharjee	2016	GPL8300 [HG_U95Av2] Affymetrix Human Genome U95 Version 2 Array	137	USA	11707567
TCGA	Eric A Collisson	2020	Illumina Hiseq	482	USA	25079552

**Table 2:** Study quality and bias in the retrospective cohort studies according to the Newcastle-Ottawa Scale (NOS) checklist.

Datasets	Total score	Cohort selection				Comparability			Outcome
		Representativeness of the Exposed Cohort	Selection of the Non-Exposed Cohort	Ascertainment of Exposure	Demonstration that the outcome of interest was not present at study initiation	Comparability of cohorts based on the design or analysis	Assessment of outcome	Was follow-up long enough for outcomes to occur	Adequacy of the follow-up of cohorts
GSE3141	7	*	*	*	*		*	*	*
GSE8894	8	*	*	*	*	*	*	*	*
GSE13213	9	*	*	*	*	**	*	*	*
GSE14814	8	*	*	*	*	*	*	*	*
GSE26939	8	*	*	*	*	*	*	*	*
GSE29013	8	*	*	*	*	*	*	*	*
GSE30219	8	*	*	*	*	*	*	*	*
GSE31210	9	*	*	*	*	**	*	*	*
GSE37745	8	*	*	*	*	*	*	*	*
GSE41271	8	*	*	*	*	*	*	*	*
GSE42127	9	*	*	*	*	**	*	*	*
GSE50081	9	*	*	*	*	**	*	*	*
GSE68465	8	*	*	*	*	*	*	*	*
GSE72094	8	*	*	*	*	*	*	*	*
GSE83227	8	*	*	*	*	*	*	*	*
TCGA	8	*	*	*	*	*	*	*	*

**Table 3:** Main results and publication bias for the meta-analysis between BUB1B and clinicopathological features, overall survival (OS), and progression-free survival (PFS).

Clinicopathological features/OS/PFS	Number of included datasets	SMD/HR(95%CI)	Z, *p* value	Heterogeneity test (I^2^, *p* value)	Publication bias (Begg’s test) (Z, *p* value)	Pooling model
Age (<=65/>65)	14	0.109 (0.028, 0.190)	2.63, 0.009	0%, 0.510	0.44, 0.661	Fixed
Sex (Male/Female)	15	0.198 (0.133, 0.262)	6.01, <0.001	43.3%, 0.058	0.69, 0.488	Fixed
Smoking status (Yes/No)	10	0.428 (0.310, 0.546)	7.11, <0.001	31.5%, 0.157	0.36, 0.721	Fixed
T stage (T2/T1)	6	0.428 (0.310, 0.545)	7.14, <0.001	37.2%, 0.158	1.88, 0.060	Fixed
T stage (T3-T4/T1-T2)	4	0.295 (0.124, 0.466)	3.38, 0.001	43.6%, 0.150	0.34, 0.734	Fixed
N stage (N1/N0)	6	0.199 (0.057, 0.341)	2.74, 0.006	26.8%, 0.233	1.13, 0.260	Fixed
N stage (N2-N3/N0-N1)	4	0.183 (0.011, 0.355)	2.09, 0.037	0%, 0.459	0.34, 0.734	Fixed
M stage (M1/M0)	5	0.293 (0.036, 0.550)	2.24, 0.025	7.4%, 0.365	-0.24, 1.000	Fixed
AJCC stage (II/I)	12	0.287 (0.178, 0.396)	5.15, <0.001	0%, 0.776	0.89, 0.373	Fixed
AJCC stage (III-IV/I-II)	9	0.126 (0.008,0.243)	2.10, 0.036	0%, 0.989	1.77, 0.067	Fixed
OS	15	1.263 (1.187, 1.340)	32.41, <0.001	39.4%, 0.058	0.59, 0.553	Fixed
PFS	11	1.243 (1.150, 1.336)	26.17, <0.001	32.2%, 0.142	0.31, 0.755	Fixed

## APPENDIX

**Table S1:** The clinical characteristics of included datasets.

Datasets	Age	Sex		Smoking history		T stage				N stage				M stage		AJCC stage			
		Female	Male	No	Yes	T1	T2	T3	T4	N0	N1	N2	N3	M0	M1	I	II	III	IV
GSE3141	-	-	-	-	-	-	-	-	-	-	-	-	-	-	-	-	-	-	-
GSE8894	59.41 ±10.40	28	33	-	-	-	-	-	-	-	-	-	-	-	-	-	-	-	-
GSE13213	60.68± 10.16	57	60	56	61	54	50	8	5	87	8	22	0	117	0	79	13	25	0
GSE14814	59.03 ±9.43	34	37	-	-	-	-	-	-	-	-	-	-	-	-	42	29	0	0
GSE26939	64.04 ±10.88	63	53	12	101	-	-	-	-	-	-	-	-	100	2	62	19	19	2
GSE29013	63.90 ±9.69	10	20	2	28	-	-	-	-	-	-	-	-	-	-	16	6	8	0
GSE30219	61.49 ±9.28	19	66	-	-	71	12	2	0	82	3	0	0	85	0	-	-	-	-
GSE31210	59.58 ±7.40	121	105	115	111	-	-	-	-	-	-	-	-	-	-	168	58	0	0
GSE37745	62.94 ±9.22	60	46	-	-	-	-	-	-	-	-	-	-	102	4	70	19	13	4
GSE41271	-	90	93	26	156	-	-	-	-	-	-	-	-	98	5	101	28	49	5
GSE42127	65.76 ±10.29	65	68	-	-	-	-	-	-	-	-	-	-	133	2	89	22	20	2
GSE50081	68.72 ±9.71	62	65	23	102	43	82	2	0	94	33	0	0	127	0	92	35	0	0
GSE68465	64.42 ±10.10	220	223	49	300	150	251	28	12	299	88	53	0	-	-	-	-	-	-
GSE72094	69.30 ±9.33	240	202	33	334	-	-	-	-	-	-	-	-	369	17	264	69	63	17
GSE83227	63.09 ±10.08	81	56	17	115	42	68	9	3	75	22	6	1	122	15	85	25	11	15
TCGA	65.10 ±10.02	258	224	64	418	165	254	45	18	319	92	68	3	461	21	263	118	80	21

**Table S2:** The results of multivariate Cox regression analysis.

Variable	HR	95%HR lower	OS			PFS		
			95% HR higher	P value	HR	95%HR lower	95%HR higher	p-value
Age	1.020	1.003	1.038	0.022	1.009	0.994	1.025	0.236
Sex (Female/Male)	0.969	0.695	1.350	0.851	0.928	0.683	1.262	0.634
Race (White/Others)	1.017	0.682	1.517	0.935	0.907	0.616	1.336	0.621
T stage	1.334	1.085	1.641	0.006	1.281	1.042	1.574	0.019
N stage	1.521	1.242	1.862	0.000	0.891	0.722	1.099	0.281
M stage	1.565	0.792	3.093	0.198	1.102	0.501	2.425	0.809
Chemotherapy (Yes/No)	0.789	0.541	1.150	0.217	1.833	1.305	2.575	<0.001
Radiotherapy (Yes/No)	2.018	1.348	3.020	0.001	3.245	2.294	4.590	<0.001
Smoking history (No/Yes)	0.992	0.616	1.599	0.974	1.149	0.735	1.797	0.542
ZWINT expression level (Low/High)	1.601	1.147	2.234	0.006	1.485	1.079	2.045	0.015
